# Dyslipidemia is associated with a poor prognosis of breast cancer in patients receiving neoadjuvant chemotherapy

**DOI:** 10.1186/s12885-023-10683-y

**Published:** 2023-03-04

**Authors:** Youzhao Ma, Minhao Lv, Peng Yuan, Xiuchun Chen, Zhenzhen Liu

**Affiliations:** Department of Breast Disease, Henan Breast Cancer Center, The Affiliated Cancer Hospital of Zhengzhou University & Henan Cancer Hospital, No.127, Dongming Road, Zhengzhou, 450008 China

**Keywords:** Breast cancer, Dyslipidemia, Pathological complete response, Neoadjuvant therapy, Disease-free survival

## Abstract

**Background:**

Lipid metabolism disorders may be involved in the occurrence and development of breast cancer. This study aimed to investigate the serum lipid changes during neoadjuvant chemotherapy for breast cancer and the effect of dyslipidemia on the prognosis of breast cancer patients.

**Methods:**

We collected the data from 312 breast cancer patients who underwent surgery after receiving standard neoadjuvant therapy. χ^2^ test and T-test were employed to analyze the effect of chemotherapy on the serum lipid metabolism of patients. The effects of dyslipidemia on the disease-free survival (DFS) of patients with breast cancer were analyzed by χ^2^ test and COX regression analysis.

**Results:**

A total of 56 out of 312 patients (17.9%) suffered from relapse. The baseline serum lipid level of the patients was significantly correlated with their age and body mass index (BMI) (*p <* 0.05). Chemotherapy increased the levels of triglycerides, total cholesterol, and low-density lipoprotein cholesterol, but decreased the level of high-density lipoprotein cholesterol (*p <* 0.001). Preoperative dyslipidemia was significantly associated with the axillary pCR rate (*p <* 0.05). COX regression analysis revealed that the full-course serum lipid level (HR = 1.896 [95%CI 1.069–3.360]; *p* = 0.029), N stage (HR = 4.416 [95%CI 2.348–8.308]; *p <* 0.001) and the total pCR rate (HR = 4.319 [95%CI 1.029–18.135]; *p* = 0.046) acted as prognostic factors affecting DFS in breast cancer. The relapse rate in patients with a high level of total cholesterol was higher than that in patients with a high level of triglycerides (61.9% vs 30.0%; *p <* 0.05).

**Conclusions:**

Dyslipidemia deteriorated after chemotherapy. The full-course serum lipid level may thus serve as a blood marker for predicting breast cancer prognosis. Serum lipids should therefore be closely monitored in breast cancer patients throughout the treatment course, and patients with dyslipidemia should be treated in a timely manner.

## Background

Breast cancer has the highest incidence rate among cases of female malignant tumors in China, accounting for approximately 69,500 deaths annually [1]. Presently, the management system for breast cancer has not been well-established in Chinese society. The problems related to the poor management of breast cancer mainly include poor control of the accompanying diseases (such as hypertension, hyperglycemia, and dyslipidemia). Elevated low-density lipoprotein cholesterol (LDL-C) is an important risk factor for cardiovascular diseases. Cardiovascular deaths in breast cancer patients account for 16.3% of all deaths [2]. Therefore, early identification and control of lipid-related risk factors during the treatment of breast cancer may help improve the prognosis of patients.

Previous studies have shown that the incidence of dyslipidemia in breast cancer patients is higher than that in healthy people [3, 4]. Abnormal lipid metabolism is considered to be related to the occurrence and progression of breast cancer, and the potential mechanism is known to mainly affect the proliferation and apoptosis of cancer cells [5]. Some studies have shown that dyslipidemia is a high risk factor for the poor prognosis of breast cancer [6, 7]. A past study reported that dyslipidemia before neoadjuvant chemotherapy affected the pathological complete response (pCR) rate of breast cancer [8]. However, other studies have suggested that baseline dyslipidemia is a good prognostic factor for breast cancer [9, 10]. The effect of dyslipidemia on the prognosis of breast cancer remains controversial. Most of these previous studies focus on the serum lipid level at a certain cut-off time, while ignoring the impact of the full-course lipid level on the prognosis of breast cancer.

Previous studies suggest that dyslipidemia deteriorated after postoperative chemotherapy in breast cancer patients [11, 12]. Moreover, the effect of chemotherapy on the deterioration of dyslipidemia is long-lasting [13]. In addition, dyslipidemia worsens after neoadjuvant chemotherapy [14]. Neoadjuvant chemotherapy is an important treatment method and a screening platform for breast cancer patients with poor prognosis; patients with non-pCR usually have a poor prognosis, and deteriorated dyslipidemia may further worsen the prognosis of non-pCR patients. However, the reported effects of different chemotherapy regimens on dyslipidemia are not consistent [11, 15]. Therefore, during chemotherapy-related decision-making, it is necessary to consider the effects of different chemotherapy schemes on lipid metabolism, which warrants accurate reference information.

Therefore, this retrospective study collected the serum lipid data of patients with breast cancer during the entire neoadjuvant therapy and analyzed the effect of the chemotherapy regimen on the serum lipid level, the correlation between the serum lipid level and clinicopathological features, and the effect of the serum lipid level on pCR and disease-free survival (DFS).

## Methods

### Patient selection

We retrospectively analyzed the clinical outcomes of 312 patients with primary breast cancer who were treated at the Henan Cancer Hospital from July 1, 2017 to May 31, 2018.

The study inclusion criteria included the following: (1) female gender; (2) invasive breast cancer with clear estrogen receptor (ER), progesterone receptor (PR), human epidermal growth factor receptor 2 (HER2), and Ki-67 status; (3) patients who had not received any drugs (including statins, phenoxyaromatic acids, niacin drugs, and cholesterol-absorption inhibitors) or treatments (including portal vena cava shunt and terminal ileectomy) that could affect the serum lipid levels within a month before baseline serum sample collection; (4) patient at stages II–III of breast cancer (AJCC 7^th^ edition); (5) all patients had reiceived neoadjuvant therapy followed by surgery, whose postoperative pathological information was available; (6) patients whose complete follow-up data was available.

The subject exclusion criteria were as follows: (1) patients with bilateral breast cancer; (2) those with advanced breast cancer; (3) those with the presence of other primary tumors in combination; (4) those with inflammatory breast cancer; (5) those with other systemic diseases that could not tolerate chemotherapy; (6) and/or those with incomplete chemotherapy cycle.

All patients underwent surgery after the completion of neoadjuvant therapy. Radiotherapy or endocrine therapy was conducted in accordance with the clinicopathological characteristics of the patients.

### Information collection and follow-up

The data on the clinicopathological features were collected, which included the age, height, weight, menstrual status, T stage, N stage, breast cancer molecular type, chemotherapy regimen, date of surgery, postoperative pathology, whether radiotherapy was applied, recurrence time, recurrence site, the baseline serum lipid level, the preoperative serum lipid levels, and the serum lipid levels from the time of treatment until the end of follow-up. The cut-off time for follow-up was June 30, 2022.

In this study, the status of ER, PR, HER2, and Ki-67 were detected by IHC conducted at the Pathology Department of our hospital. The HR positivity criteria were ER ≥ 1% or PR ≥ 1%; HER2-positive standard: IHC detection of HER2 was 3 + or 2 + , fluorescence *in situ* hybridization (FISH) detection was *HER-2* amplification (HER2/chromosome enumeration probe 17 [CEP17] ratio ≥ 2.0, average HER2 copy number ≥ 4.0 signals per cell; or HER2/CEP17 ratio < 2.0, average HER2 copy number ≥ 6.0 signals per cell). While Ki-67 cut-off criteria varied across the centers, we considered a sample to be Ki-67-high if the proliferation index was > 14%. T staging was confirmed via ultrasound or magnetic resonance imaging (MRI); N staging with positive palpation or suspicious imaging was confirmed through fine needle biopsy or core needle biopsy (AJCC 7^th^ edition). PCR was defined as the absence of residual tumor cells in the primary tumor (breast pCR) and axillary lymph nodes (axillary pCR) after neoadjuvant chemotherapy (ypT0ypN0). DFS was defined as the period from surgery to disease relapse or death from any cause. We collected data on the postoperative recurrence, metastasis information, and DFS of all patients.

### Data collection and evaluated parameters

The serum lipid levels were measured before the treatment, before surgery, and at each follow-up examination after surgery. The automatic biochemical analyzer in our hospital was used to measure the concentration of total cholesterol (TC), triglyceride (TG), low-density lipoprotein-cholesterol (LDL-C), and high-density lipoprotein-cholesterol (HDL-C) in the serum of the patients.

The criteria for determining dyslipidemia were referred from those prescribed by the Joint Committee for the Development of Chinese Adult Dyslipidemia Prevention and Control Guidelines [16], as follows: serum TGs ≥ 2.26 mmol/L; serum TC ≥ 6.22 mmol/L; serum LDL-C ≥ 4.14 mmol/L; serum HDL-C < 1.04 mmol/L. The full-course dyslipidemia was defined as the detection of dyslipidemia thrice or more during the whole study process. As body mass index (BMI) could be related to the serum lipid levels, we assigned the patients into 3 groups based on their body mass index (BMI). BMI was calculated using the following formula: BMI = weight (kg)/height*height (m^2^). BMI grading was performed in accordance with the World Health Organization's grading scale.

### Chemotherapy regimens

The neoadjuvant chemotherapy regimens in this study were conducted as follows:

1. TEC (docetaxel 75 mg/m^2^, epirubicin 75 mg/m^2^, and cyclophosphamide 500 mg/m^2^,, every 3 weeks for 6 cycles).
2. EC-T/EC-TH (epirubicin 90 mg/m^2^ and cyclophosphamide 600 mg/m^2^ every 3 weeks for 4 cycles, followed by docetaxel 100 mg/m^2^ and/or trastuzumab 8 mg/kg loading dose, 6 mg/kg maintenance dose, every 3 weeks for 4 cycles).
3. TCH (docetaxel 75 mg/m^2^, carboplatin AUC 6, and trastuzumab 8 mg/kg loading dose, 6 mg/kg maintenance dose, every 3 weeks for 6 cycles).
4. TC (docetaxel 75 mg/m^2^ and cyclophosphamide 600 mg/m^2^, every 3 weeks for 4 cycles).

TEC, EC-T, and EC-TH are taxane-plus-anthracyclines-based regimens; TC and TCH are taxane-based regimens.

### Statistical analysis

The SPSS 23.0 was used for statistical analyses. The correlation between the serum lipids level and the clinical data was analyzed by the χ^2^ test, with *p <* 0.05 considered as the significance threshold. Univariate analysis of factors associated with recurrence and metastasis was evaluated by the χ^2^ test. Independent risk factors affecting prognosis were analyzed by multivariate COX regression. Factors with *p <* 0.05 in the COX regression analysis were considered independent prognostic factors. The Kaplan–Meier survival curve was employed to reflect the effect of serum lipid levels on survival at different time points.

## Results

### The relationship between the baseline serum lipid levels and clinicopathological characteristics

A total of 312 breast cancer patients were enrolled in this study. These patients were of a median age of 48 years (age range: 26–74 years). There were 256 (83.7%) breast cancer patients with baseline normal serum lipids, and 50 (16.3%) patients with baseline dyslipidemia. Compared with patients with normal serum lipids, those with dyslipidemia were more likely to be older (*p* = 0.025) and had higher BMI (*p* = 0.047). The baseline serum lipid level was not associated with menopausal status, cT stage, cN stage, HR status, HER2 status, Ki-67, and molecular subtype (Table 1).

**Table 1:** Characteristics of the patients according to the baseline lipid levels

Characteristic	Total	Baseline serum lipids		*P* value
		Normal	Dyslipidemia	
		256(83.7)	50(16.3)	
Age(years)				**0.025**
≤ 35	37	35 (94.6)	2 (5.4)	
35 ~ 55	197	167 (84.8)	30 (15.2)	
> 55	72	54 (75.0)	18 (25.0)	
Menopausal				0.264
Premenopausal	216	184 (85.2)	32 (14.8)	
Postmenopausal	90	72 (80.0)	18 (20.0)	
BMI				**0.047**
< 24	125	113 (90.4)	12 (9.6)	
24 ~ 28	131	106 (80.9)	25 (19.1)	
> 28	45	35 (77.8)	10 (22.2)	
cT				0.873
T1	34	30 (88.2)	4 (11.8)	
T2	212	177 (83.5)	35 (16.5)	
T3	38	31 (81.6)	7 (18.4)	
T4	22	18 (81.8)	4 (18.2)	
cN				0.662
N0 ~ 1	198	167 (84.3)	31 (15.7)	
N2 ~ 3	108	89 (82.4)	19 (17.6)	
HR				0.430
Negative	113	97 (85.5)	16 (14.2)	
Positive	193	159 (82.4)	34 (17.6)	
HER2				0.289
Negative	194	159 (82.0)	35 (18.0)	
Positive	112	97 (86.6)	15 (13.4)	
Ki-67				0.750
≤ 14%	10	8 (80.0)	2 (20.0)	
> 14%	296	248 (83.8)	48 (16.2)	
Subtype				0.703
HR + /HER2-	130	105 (80.8)	25 (19.2)	
HR + /HER2 +	62	53 (85.5)	9 (14.5)	
HR-/HER2 +	52	45 (86.5)	7 (13.5)	
HR-/HER2-	62	53 (85.5)	9 (14.5)	

*BMI* Body mass index, *HR* Hormone receptor, *HER2* Human epidermal growth factor receptor 2, + Positive, − Negative

### The effects of chemotherapy on the serum lipid levels

**Fig. 1:**
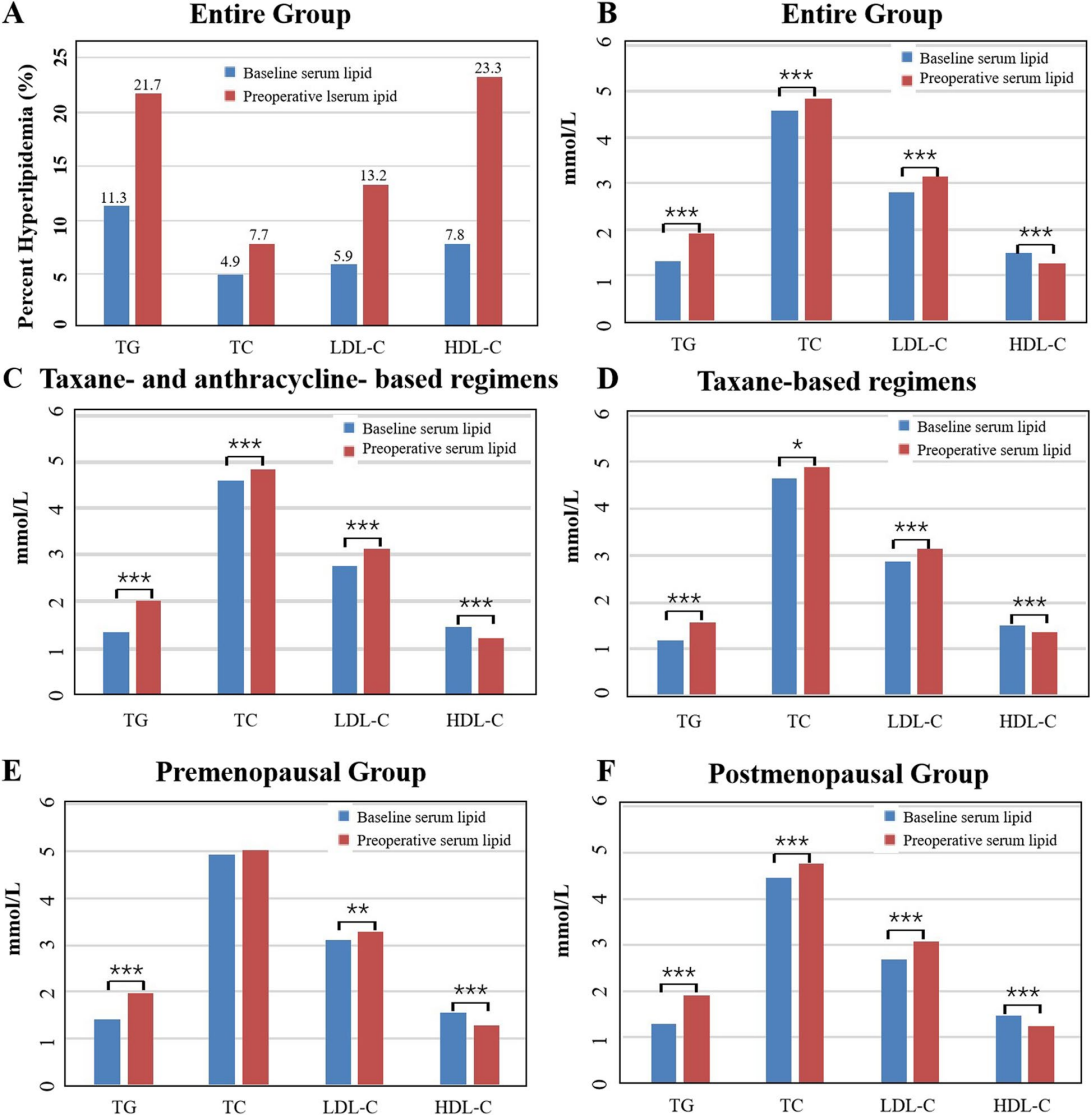
Effects of neoadjuvant chemotherapy on serum lipids. **A** and **B**: Changes of serum lipids before and after neoadjuvant chemotherapy. **C** and **D**: Effects of different chemotherapy regimens on serum lipids. **E** and **F**: Effects of chemotherapy on serum lipids in different menopausal status

The present results indicated an increased incidence of high TC, high TG, high LDL-C, and low HDL-C in the serum of patients after chemotherapy (Fig. 1A). The whole population showed a significant increase in the TG (*P <* 0.001), TC (*p <* 0.001), and LDL-C (*p <* 0.001) values as well as a significant decrease in the HDL-C (*p <* 0.001) value after chemotherapy (Table 2; Fig. 1B).

**Table 2:** Comparison of the serum lipid profiles pre- and post-chemotherapy

Parameters	Baseline serum lipids	Preoperative serum lipids	*P* value
Entire group (*n* = 295)			
TG	1.32 ± 0.99	1.92 ± 1.74	< 0.001
TC	4.60 ± 1.01	4.84 ± 1.11	< 0.001
LDL-C	2.81 ± 0.83	3.14 ± 0.90	< 0.001
HDL-C	1.48 ± 0.40	1.26 ± 0.32	< 0.001
Taxane-and anthracycline-based regimens(*n* = 227)			
TG	1.36 ± 1.08	2.02 ± 1.93	< 0.001
TC	4.59 ± 1.01	4.83 ± 1.15	< 0.001
LDL-C	2.79 ± 0.83	3.14 ± 0.91	< 0.001
HDL-C	1.47 ± 0.40	1.23 ± 0.32	< 0.001
Taxane-based regimens(*n* = 68)			
TG	1.20 ± 0.59	1.59 ± 0.74	< 0.001
TC	4.66 ± 1.01	4.88 ± 0.98	0.05
LDL-C	2.89 ± 0.84	3.15 ± 0.90	0.005
HDL-C	1.53 ± 0.36	1.36 ± 0.30	< 0.001
Pre-menopause(*n* = 87)			
TG	1.41 ± 0.74	1.96 ± 1.92	0.04
TC	4.94 ± 0.92	5.02 ± 0.98	0.415
LDL-C	3.11 ± 0.79	3.30 ± 0.91	0.028
HDL-C	1.54 ± 0.41	1.28 ± 0.32	< 0.001
Postmenopause (*n* = 208)			
TG	1.29 ± 1.07	1.90 ± 1.66	< 0.001
TC	4.46 ± 1.02	4.77 ± 1.16	< 0.001
LDL-C	2.69 ± 0.81	3.08 ± 0.89	< 0.001
HDL-C	1.46 ± 0.39	1.25 ± 0.32	< 0.001

*TG* Triglycerides, *TC* Total cholesterol, *LDL-C* Low-density lipoprotein cholesterol, *HDL-C* High-density lipoprotein cholesterol

To assess the chemotherapy regimens on serum lipids, the participants were categorized into 2 groups: taxane- plus anthracyclines-based regimens and taxane-based regimens. Subgroup analysis of the chemotherapy regimens revealed that both the chemotherapy regimens caused an increase in the TC, TG, and LDL-C values and a decrease in the HDL-C value (Fig. 1C and D). The analysis of the menstrual status indicated that TG (*p* = 0.04) and LDL-C (*p* = 0.028) had increased, while HDL-C (*p <* 0.001) had decreased after chemotherapy in the premenopausal patients, albeit the difference in the TC value was not significant (Fig. 1E). In postmenopausal patients, chemotherapy led to an increase in the values of TG, TC, and LDL-C and a decrease in the value of HDL-C (*p <* 0.001) (Fig. 1F).

### Correlations between the serum lipid levels and pCR

To explore the relationship between the serum lipid levels and chemotherapy sensitivity, we further analyzed the correlation between the serum lipid levels and pCR after administering neoadjuvant chemotherapy. The preoperative lipid levels were significantly associated with the axillary pCR rates (*p <* 0.05); the rate of axillary pCR in patients with normal serum lipids was significantly higher than that in patients with dyslipidemia. However, no significant correlation was noted between the baseline serum lipid levels and the pCR rates in this study (Table 3).

**Table 3:** Analysis of the correlation between serum lipid levels and pCR

Characteristic	Baseline serum lipids		χ^2^	*P*	Preoperative serum lipids		χ^2^	*P*
	Normal	Dyslipidemia			Normal	Dyslipidemia		
Total pCR			0.000	0.990			3.358	0.067
No	205 (80.1)	40 (80.0)			154 (76.6)	84 (85.7)		
Yes	51 (19.9)	10 (20.0)			47 (23.4)	14 (14.3)		
Breast pCR			0.640	0.424			0.832	0.362
No	193 (75.4)	35 (70.0)			146 (72.6)	76 (77.6)		
Yes	63 (24.6)	15 (30.0)			55 (27.4)	22 (22.4)		
Axillary pCR			2.413	0.120			6.764	**0.009**
No	128 (50.0)	31 (62.0)			95 (47.3)	62 (63.3)		
Yes	128 (50.0)	19 (38.0)			106 (52.7)	36 (36.7)		

*pCR* Pathological complete response

### Dyslipidemia prompts poor prognosis in NAC breast cancer patients

**Fig. 2:**
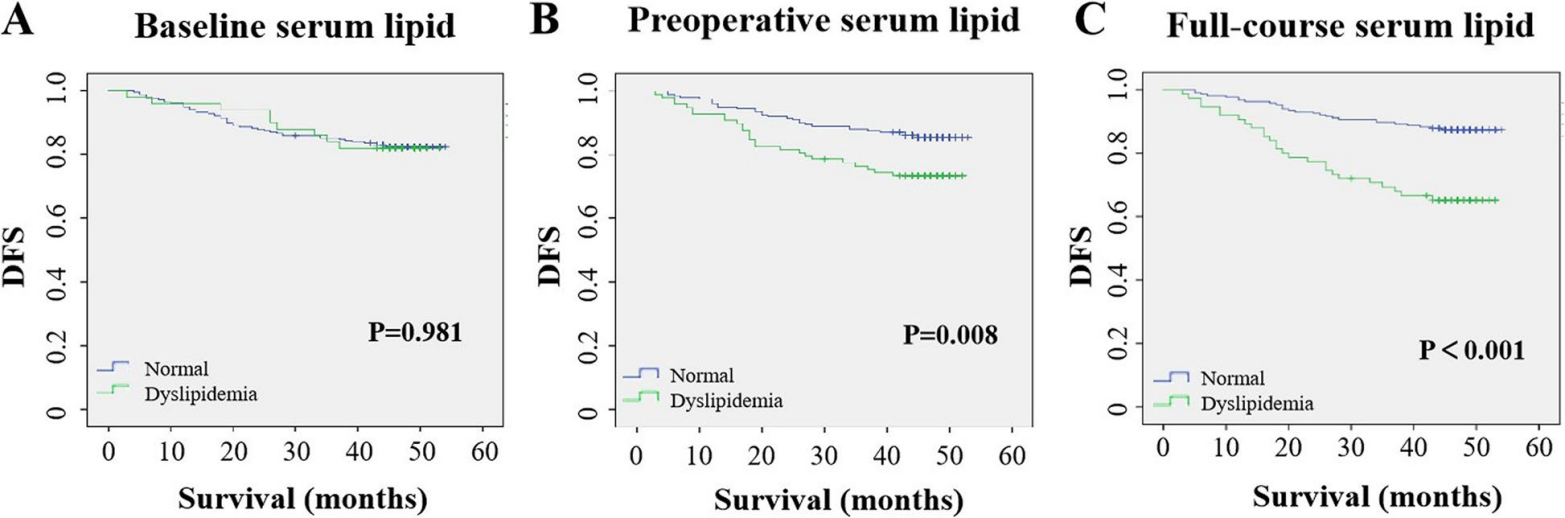
Kaplan–Meier curves for DFS in the whole population according to baseline serum lipid, preoperative serum lipid and full-course serum lipid levels. DFS: disease-free survival

During a median follow-up of 47 months (range: 3–54 months), 56 patients (17.9%) suffered from relapse. The Kaplan–Meier survival curve indicated that the baseline serum lipid level had no significant impact on DFS, and the DFS was worse in patients with preoperative (*p* = 0.008) and full-course dyslipidemia (*p <* 0.001) (Fig. 2).

Univariate and COX regression analyses showed that the full-course serum lipid level (HR = 1.896 [95%CI 1.069–3.360]; *p* = 0.029), N stage (HR = 4.416 [95%CI 2.348–8.308]; *p <* 0.001), and total pCR rate (HR = 4.319 [95%CI 1.029–18.135]; *p* = 0.046) were the prognostic factors affecting DFS in breast cancer (Table 4).

**Table 4:** Prognostic factors for disease-free survival

Characteristic	Total	No-Relapse *n* (%)	Relapse *n* (%)	Univariate analysis		Multivariate analysis		
				χ^2^	*P*	HR	95%CI	*P*
Age(years)				10.359	**0.006**			0.209
< 35	37	28 (75.7)	9 (24.3)			Ref	Ref	
35–55	202	176 (87.1)	26 (12.9)			0.661	0.305–1.434	0.295
> 55	73	52 (71.2)	21 (28.8)			1.124	0.490–2.580	0.783
Menopausal				1.569	0.210			
Premenopausa	222	186 (83.8)	36 (16.2)					
Postmenopausal	90	70 (77.8)	20 (22.2)					
BMI				2.470	0.291			
< 24	126	106 (84.1)	20 (15.9)					
24 ~ 28	134	105 (78.4)	29 (21.6)					
> 28	47	41 (87.2)	6 (12.8)					
Full-course serum lipid				18.030	** < 0.001**			
Normal	214	187 (87.4)	27 (12.6)			Ref	Ref	
Dyslipidemia	75	49 (65.3)	26 (34.7)			1.896	1.069–3.360	**0.029**
cT				6.454	**0.011**			
T1 ~ 2	250	212 (84.8)	38 (15.2)			Ref	Ref	
T3 ~ 4	62	44 (71.0)	18 (29.0)			1.506	0.807–2.811	0.198
cN				47.228	** < 0.001**			
N0 ~ 1	202	188 (93.1)	14 (6.9)			Ref	Ref	
N2 ~ 3	110	68 (61.8)	42 (38.2)			4.416	2.348–8.308	** < 0.001**
Radiotherapy				1.159	0.282			
No	37	28 (75.7)	9 (24.3)					
Yes	275	228 (82.9)	47 (17.1)					
HR				0.278	0.598			
Negative	113	91 (80.5)	22 (19.5)					
Positive	199	165 (82.9)	34 (17.1)					
HER2				0.003	0.956			
Negative	196	161 (82.1)	35 (17.9)					
Positive	116	95 (81.9)	21 (18.1)					
Ki-67				0.03	0.864			
≤ 14%	10	8 (80.0)	2 (20.0)					
> 14%	302	248 (82.1)	54 (17.9)					
Subtype				1.466	0.690			
HR + /HER2-	132	111 (84.1)	21 (15.9)					
HR + /HER2 +	66	52 (78.8)	14 (21.2)					
HR-/HER2 +	52	44 (84.6)	8 (15.4)					
HR-/HER2-	62	49 (79.0)	13 (21.0)					
Total pCR				11.389	**0.001**			
Yes	62	60 (96.8)	2 (3.2)			Ref	Ref	
No	250	196 (78.4)	54 (21.6)			4.319	1.029–18.135	**0.046**

*BMI* Body mass index, *HR* Hormone receptor, *HER2* Human epidermal growth factor receptor 2, + Positive, − Negative

**Fig. 3:**
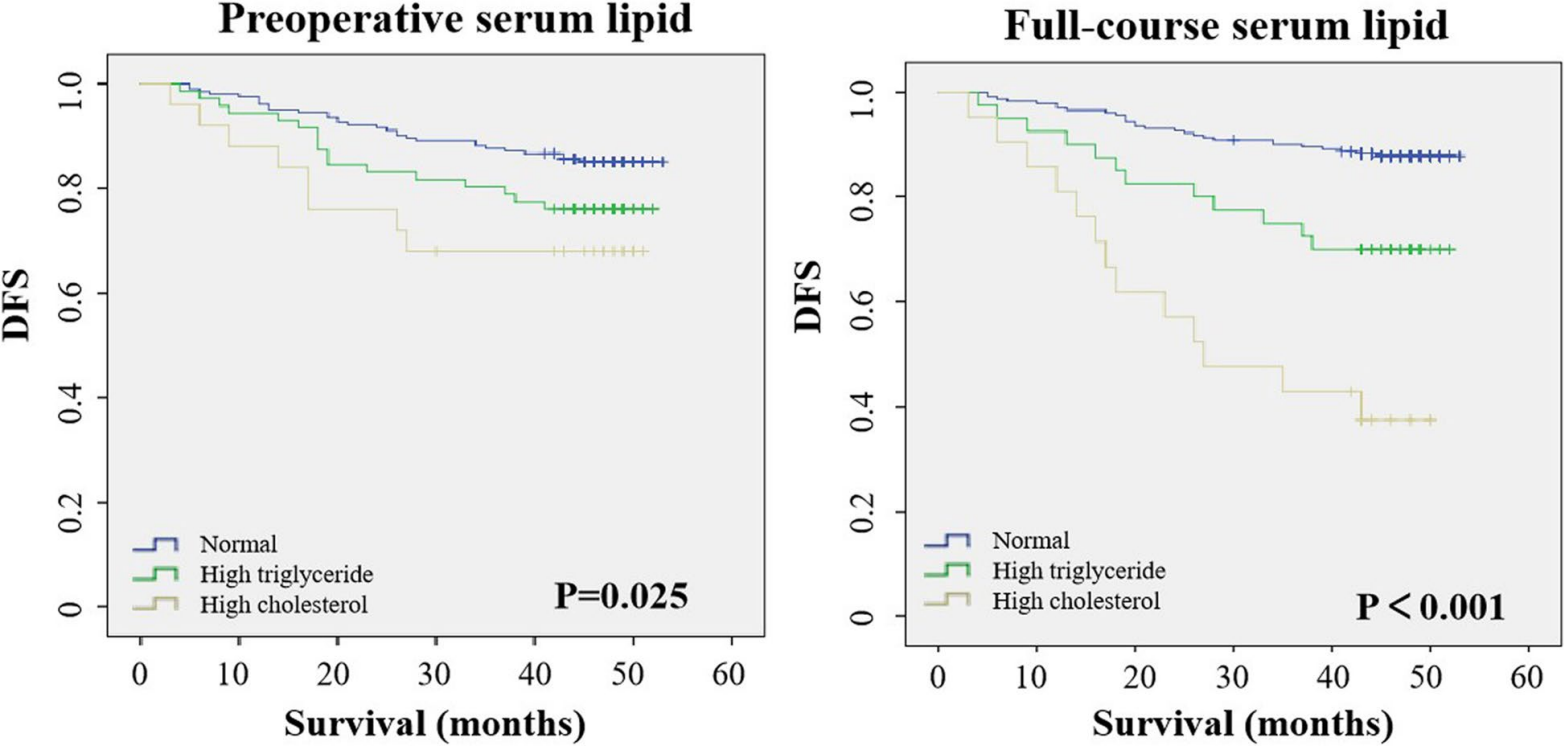
Kaplan–Meier curves for DFS in the whole population according to preoperative TG, TC and full-course TG, TC levels. DFS: disease-free survival; TG: triglyceride; TC: total cholesterol

**Fig. 4:**
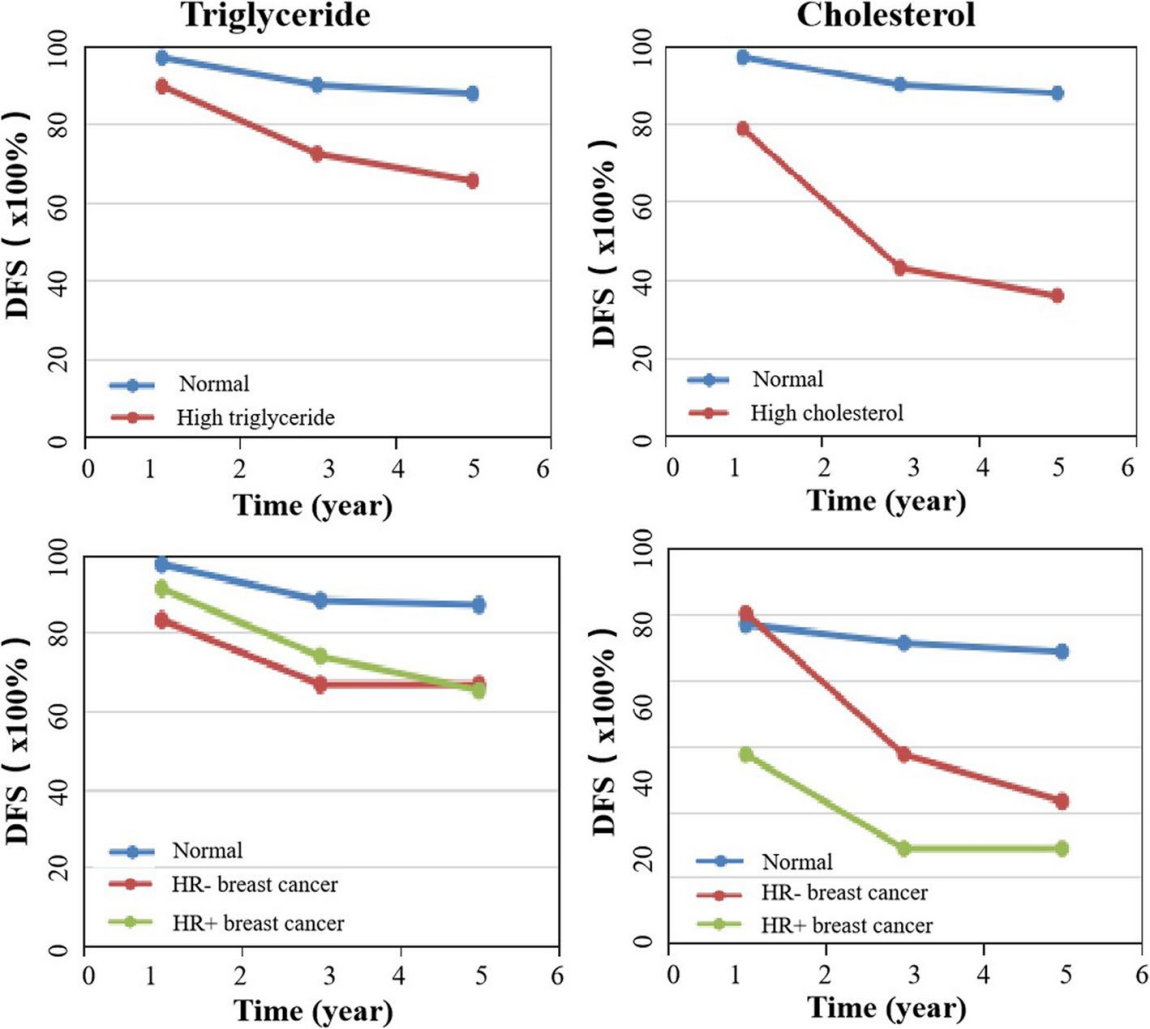
The 1-,3-,5-year DFS in HR- and HR + breast cancer patients according to triglyceride and total cholesterol. **A** and **B**: DFS in high triglyceride and high total cholesterol breast cancer patients. **C** and **D**: Effects of high triglycerides and high total cholesterol on DFS in HR- and HR + breast cancer patients. DFS: disease-free survival; HR: hormone receptor

To further clarify the specific serum lipid indicators that affected breast cancer DFS, we analyzed the full-course TG and TC levels. The results revealed that patients with a high full-course TC level had a higher relapse rate than those with a high level of TG (61.9% vs 30.0%; *p <* 0.05) (Table 5; Fig. 3). Comparison of the 1-, 3-, and 5-year DFS rates of patients with different serum lipid levels revealed that the 1-, 3-, and 5-year DFS rates of patients with hyperlipidemia were lower than those of patients with normal serum lipids. Next, we analyzed the prognostic role of the lipid levels in HR-positive and negative patient populations. The results revealed that the DFS of hypercholesterolemic patients in HR-positive patients was significantly lower than that in HR-negative patients (Fig. 4).

**Table 5:** Analysis of the correlation between high triglyceride / high cholesterol and relapse

Characteristic	Total	No-Relapse *n* (%)	Relapse *n* (%)	Univariate analysis	
				χ^2^	*P*
Full-course serum lipid				36.274	** < 0.001**
Normal	230 (79.0)	202 (87.8)	28 (12.2)		
High triglyceride	40 (13.8)	28 (70.0)	12 (30.0)		
High cholesterol	21 (7.2)	8 (38.1)	13 (61.9)		
Preoperative serum lipid				6.212	0.045
Normal	202 (70.6)	172 (85.1)	30 (14.9)		
High triglyceride	62 (21.7)	54 (76.1)	17 (23.9)		
High cholesterol	22 (7.7)	17 (68.0)	8 (32.0)		
Baseline serum lipid				0.064	0.968
Normal	256 (83.7)	211 (82.4)	45 (17.6)		
High triglyceride	35 (11.3)	29 (82.9)	6 (17.1)		
High cholesterol	15 (4.9)	12 (80.0)	3 (20.0)

Finally, we analyzed the effect of endocrine drugs on serum lipid levels. Among the patients undergoing endocrine therapy after surgery in this study (*n* = 154), the incidence of serum dyslipidemia in the 3 groups of letrozole/anastrozole, tamoxifen/toremifene, and exemestane, respectively, were 28.7%, 21.3%, and 16.7% (*p* = 0.509).

## Discussion

Breast cancer is the most commonly diagnosed cancer in women and the leading cause of cancer death [17]. The incidence of dyslipidemia in breast cancer patients is higher than that in healthy people [3, 4]. Both the impact of chemotherapy on serum lipids and the impact of serum lipids on prognosis are controversial [6–11, 15]. Neoadjuvant chemotherapy provides a choice of intensive treatment for non-pCR patients. Meanwhile, neoadjuvant chemotherapy is a good experimental platform, which can screen out the prognostic factors of breast cancer. Therefore, we collected clinicopathological data of patients receiving neoadjuvant therapy and conducted a series of analyses on the relationship between dyslipidemia and clinicopathological features, the effect of chemotherapy on serum lipids, and the effect of serum lipids on cancer prognosis.

Currently, chemotherapy remains one of the most important treatment methods for breast cancer. While killing tumor cells, chemotherapeutic drugs can also affect other body indicators of the patients, which includes normal blood cells, liver functions, and serum lipids. Serum lipids are the collective term used to mainly refer to TC, TG, HDL-C, and LDL-C. Previous studies have shown that the levels of TG, TC and LDL-C in breast cancer patients significantly increased after chemotherapy, while the levels of HDL-C decreased [12, 15, 18, 19]. Paclitaxel-based chemotherapy significantly worsens dyslipidemia [20], while anthracycline-based chemotherapy has a slight impact on the deterioration of dyslipidemia [11]. However, a study found that anthracycline drugs significantly lower HDL-C levels than taxanes [15]. Briefly, the effect of different chemotherapy regimens on serum lipids is controversial. In our study, both the taxane-plus anthracycline-based regimens and the taxane-based regimens led to an increase in the levels of TC, TG, and LDL-C, but a decrease in the level of HDL-C. This finding is consistent with the results of previous studies. Therefore, chemotherapy patients need to closely monitor for any change in their serum lipid levels to achieve good control.

Numerous pieces of evidence indicate that changes in lipid metabolism can affect cancer cell proliferation, differentiation, and other processes [21]. In animal experiments, dyslipidemia can promote the progress of breast cancer cells [22, 23]. Previous clinical studies also reported that dyslipidemia is a high risk factor for the poor prognosis of breast cancer [6, 7]. However, some studies have reported that baseline dyslipidemia is a good prognostic factor for breast cancer [9, 10]. Interestingly, some studies reported that statins can improve the prognosis of breast cancer [24–26]. It is unknown whether there exists a correlation between the above two findings. However, the mechanism by which statins can improve the prognosis of cancer remains unclear. In a word, the current researches on the role of serum lipids in the prognosis of breast cancer remain controversial. This controversy may be attributed to the fact that the serum lipid level at a certain time point could not be representative of the serum lipid level during the entire treatment duration. We found that preoperative dyslipidemia reduces the axillary pCR rate. In terms of prognosis, our results revealed that the baseline lipid level was not significantly associated with DFS, but the full-course lipid level was significantly associated with DFS in breast cancer patients. Therefore, we should pay close attention to the control of serum lipids during the whole course of breast cancer treatment, which can improve the prognosis of patients.

Endocrine therapy is an important subset of systemic therapy for HR + /HER2- breast cancer. Endocrine therapy drugs mainly involve tamoxifen, anastrozole, letrozole, and exemestane. The effects of different endocrine therapy drugs on serum lipid changes in previous studies are controversial. Some studies have reported that tamoxifen and exemestane reduce the risk of dyslipidemia in breast cancer patients [27–29]. However, in another study, both exemestane and letrozole treatment lead to detrimental changes in the lipid profile of postmenopausal women with breast cancer [30]. The proportion of dyslipidemia in patients taking trozole/anastrozole was numerically higher than that in patients taking tamoxifen/toremifene and exemestane in our study. The lack of statistical difference here may be attributed to the small sample size. However, the effect of endocrine drugs on specific indicators of serum lipids was not analyzed in the present study owing to the small sample size. We plan to continue exploring this issue in our future research. The choice of endocrine therapy drugs should be carefully selected based on each individual's serum lipid levels.

The strength of this study lies in that we collected and analyzed the full-course serum lipid data. This study, however, has some limitations, such as the retrospective research design. The short follow-up time may have affected the results of the prognosis analysis. In addition, our data were collected from a single center.

## Conclusions

The baseline serum lipid levels were not significantly associated with DFS in breast cancer, but the full-course lipid levels were significantly associated with DFS in breast cancer. Dyslipidemia deteriorated after both anthracycline- and taxane-based chemotherapy. Serum lipids should therefore be closely monitored in breast cancer patients throughout the course of treatment, and patients with dyslipidemia should be treated without delay.

## Data Availability

The datasets used and/or analyzed during the current study are available from the corresponding author upon reasonable request.

## Abbreviations

TC: Total cholesterol
TG: Triglycerides
LDL-C: Low-density lipoprotein cholesterol
HDL-C: High-density lipoprotein cholesterol
BMI: Body mass index
95% CI: 95% Confidence interval
DFS: Disease-free survival
HR: Hazard ratio
pCR: Pathological complete response
ER: Estrogen receptor
PR: Progesterone receptor
HER2: Human epidermal growth factor receptor 2
